# Ferroptosis and cuproptposis in kidney Diseases: dysfunction of cell metabolism

**DOI:** 10.1007/s10495-023-01928-z

**Published:** 2023-12-14

**Authors:** Tingting Chen, Lifei Liang, Yuzhu Wang, Xiaoyu Li, Cheng Yang

**Affiliations:** 1 Department of Pharmacy, Zhongshan Hospital, Fudan University, Shanghai, China; 2 Department of Urology, Zhongshan Hospital, Fudan University, No. 180 Fenglin Road, Shanghai, China; 3 Shanghai Key Laboratory of Organ Transplantation, Shanghai, China; 4 Zhangjiang Institue of Fudan University, Shanghai, China

**Keywords:** Ferroptosis, Cuproptosis, Metal ions, Cell death, Renal cancer, Kidney ischemia-reperfusion injury, Kidney fibrosis, Kidney transplantation

## Abstract

Metal ions play an important role in living organisms and are involved in essential physiological activities. However, the overload state of ions can cause excess free radicals, cell damage, and even cell death. Ferroptosis and cuproptosis are specific forms of cell death that are distinct from apoptosis, necroptosis, and other regulated cell death. These unique modalities of cell death, dependent on iron and copper, are regulated by multiple cellular metabolic pathways, including steady-state metal redox treatment mitochondrial activity of lipid, amino acid and glucose metabolism, and various signaling pathways associated with disease. Although the mechanisms of ferroptosis and cuproptosis are not yet fully understood, there is no doubt that ion overload plays a crucial act in these metal-dependent cell deaths. In this review, we discussed the core roles of ion overload in ferroptosis and cuproptosis, the association between metabolism imbalance and ferroptosis and cuproptosis, the extract the diseases caused by ion overload and current treatment modalities.

**Facts**

- Ferroptosis and cuproptosis are distinct forms of regulated programmed cell death that are characterized by their dependence on excessive accumulation of iron and copper ions, respectively.
- The oxidation state of metal ions plays a crucial role in the mechanisms of ferroptosis and cuproptosis. Specifically, Fe^2+^ promotes ferroptosis, whereas Cu^+^ is a more potent form than Cu^2+^ in promoting cuproptosis.
- Overload of iron ions can induce ferroptosis not only by triggering the Fenton reaction and causing direct peroxidation of PUFA-PIs, but also by serving as a necessary cofactor for enzymes involved in lipid peroxidation.
- Intracellular copper accumulation results in the aggregation of mitochondria lipoylated proteins and destabilization of Fe-S cluster proteins, thereby triggering cuproptosis.
- Both ferroptosis and cuproptosis affect mitochondria and exhibit crosstalk, owing to the close relationship between copper and iron metabolism.

**Open questions**

- What are the precise morphological and biochemical changes associated with cuproptosis?
- What is the exact quantitative relationship between ion overload and cell death? What is the threshold of ion overload that induces cell death?
- How does iron metabolism regulate cuproptosis? How dose copper metabolism regulate ferroptosis? What are the specific interactions between ferroptosis and cuproptosis?
- Dose the generation of reactive oxygen species (ROS) from copper-based Fenton reactions play a role in triggering ferroptosis?
- Can other trace metal ions induce cell death through mechanisms distinct from the existent forms of cell death?

## Introduction

Transition metal ions, including iron (Fe), copper (Cu), zinc (Zn), and manganese (Mn), are vital for life and are involved in many essential biochemical processes in living organisms, ranging from bacteria to human beings [1, 2]. These processes include carbon transformation, nucleic acid and protein synthesis, deoxyribonucleic acid (DNA) replication, the tricarboxylic acid (TCA) cycle, electron transport in mitochondria, glycolysis, and the metabolism of reactive oxidative species (ROS) [2, 3].

Maintaining metal ion homeostasis is crucial to achieve a proper balance of these ions in different cellular compartments. External or intrinsic alterations to metal ion metabolism can lead to ion deficiency or ion overload [1, 4]. Ion deficiency can limit ion availability for metalloenzyme synthesis and other biochemical pathways in multiple tissues, ultimately leading to reduced work capacity, developmental retardation, and various diseases such as hemochromatosis, cardiovascular disease [5, 6], iron deficiency anemia [7], Parkinson’s disease, and Friedreich’s ataxia and Pica [8–10]. Conversely, an excess of redox-active essential metals such as Fe and Cu can induce free radicals under certain conditions, causing inflammation, cell damage and cancerous changes, eventually leading to cardiovascular diseases, neurodegenerative diseases and metal ion overload disorders [11–14]. Each metal can react in a unique way and has different mechanisms of action. In parallel processes, excessive metal ions can induce oxidative stress, damage the antioxidant defense system, alter the redox balance, affect intracellular signaling pathways, and ultimately lead to cell damage or death.

Ferroptosis, driven by iron-dependent phospholipid peroxidation, has been recognized associated tightly with iron overload [15]. In addition to iron-dependent cell death, a recent study found that copper accumulation triggers the aggregation of mitochondrial lipoylated proteins and the destabilization of iron-sulfur (Fe-S) cluster proteins, leading to a particular type of cell death termed cuproptosis [16]. These metal-dependent cell death are distinctly different from apoptosis, necroptosis, and other regulated cell death (RCD) (Table 1). In this review, we discuss the central roles of iron and copper ions overload on metal-dependent cell death, summarize the regulation by multiple cellular metabolism pathways, present the interrelationship between these two forms of metal-dependent cell death and extract the diseases caused by ion overload.

**Table 1:** Morphological and biochemical features of different forms of RCD

	Morphological Features	Biochemical Features	Key Genes
Cuproptosis	NA	Lipoylated protein aggregation and iron-sulfur cluster protein loss	FDX1, ATP7A/B, DLAT
Ferroptosis	Lack of rupture and blebbing of the plasma membrane, small mitochondria with increased mitochondrial membrane densities, reduction or vanishing of mitochondria Crista, outer mitochondrial membrane Rupture and normal nucleus	Iron accumulation and lipid peroxidation	GPX4, TFR1, SLC7A11, NRF2, NCOA4, P53, HSPB1, ACSL4, FSP1
Apoptosis	Cell shrinkage, membrane blebbing, cellular and nuclear volume reduction, chromatin agglutination, nuclear fragmentation, formation of apoptotic bodies and cytoskeletal disintegration, no obvious alteration of mitochondria.	DNA fragmentation, activation of caspases, phosphatidylserine exposure	Caspase, Bcl-2, Bax, P53, Fas
Necroptpsis	Plasma membrane disruption, cell and organelle swelling, moderate chromatin condensation, and leakage of cellular constituents	Drop in ATP levels; Activation of RIPK1, RIPK3, and MLKL; cytosolic necrosome formation	RIPK1, RIPK3
Pyroptosis	Plasma membrane bubbling and cell swelling with an intact nucleus	Activation of caspase1, caspase3, and GSDMD; GSDMD cleavage; GSDMD-N–induced pore formation; IL1B release	Caspase, GSDMD
Autophagy	Normal cell membrane, formation of double-membraned autolysosomes, including macroautophagy, microautophagy and chaperone-mediated autophagy, lack of chromatin condensation.	Increased lysosomal activity (e.g., LC3-I to LC3-II conversion)	ATG5, ATG7, LC3, Beclin-1, DRAM3, TFEB

*ACSL4, acyl-CoA synthetase long-chain family member 4; ATG5, autophagy-related 5; ATG7, autophagy-related 7; Bax, BCL2-associated X protein; DLAT, dihydrolipoamide S-acetyltransferase; DRAM3, damage-regulated autophagy modulator 3; FDX1, Ferredoxin-1; FSP1, ferroptosis suppressor protein 1, GPX4, glutathione peroxidase 4; GSDMD, gasdermin D; HSPB1, heat shock protein beta-1; LC3, microtubule-associated protein 1 light chain3; IL1B, interleukin-1 β; MLKL, mixed lineage kinase domain-like; NCOA4, nuclear receptor coactivator 4; NRF2, nuclear factor erythroid 2-related factor 2; RCD, regulated cell death; RIPK, receptor-interacting serine/threonine protein kinase; SLC7A11, solute carrier family 7 member 11; TFEB, transcription factor EB; TFR1, transferrin receptor 1*

## Metal ions are deeply involved in cell metabolism

Metal ions play a vital role in cell metabolism, as they participate in various ionic states throughout processes such as absorption, distribution, storage, and elimination, at both the cellular and systemic levels in multicellular organisms.

Iron plays a crucial role in the formation and metabolism of ROS due to its ability to carry out one-electron reactions, determined by the electronic structure of the iron atom [2]. Iron is commonly found in ferric-reduced and ferrous-oxidized states, which it oscillates between under physiological conditions, and to a lesser extent in the highly oxidizing ferryl (Fe^4+^) form [2, 4]. Ferric iron (Fe^3+^) is bound to proteins, such as transferrin, to ensure good bioavailability and overcome solubility issues, as it is stable but poorly soluble in water [17]. This allows Fe^3+^ to be transported steadily in a redox-inactive state. In contrast, ferrous iron (Fe^2+^) is high reactivity and water-soluble and can generates ROS through the Fenton reaction [17]. Iron is essential for various type of biological processes in almost all forms of life, including being incorporated into multiple proteins as organic cofactors, such as iron-sulphur clusters (ISC) in matrix proteins like biotin synthase, aconitase, homoaconitase and ferredoxin [18]. Iron-containing proteins also play important roles in the catalytic centers of many enzymes, such as ribonucleotide reductase and DNA helicase during DNA replication, cytochrome oxidases in electron transport, and the TCA cycle for oxidative phosphorylation (OXPHOS) and energy production [19–22]. Iron levels are maintained within a range of 3–4 g by precise control of its absorption, mobilization, storage, and recycling in healthy individuals [23]. Given the importance of iron in fundamental physiological processes, sufficient iron is necessary for cell metabolism and development.

Copper is a crucial trace metal micronutrient that plays an essential role in various cell metabolism processes in mammals. Despite its lower abundance compared to other metals like iron and zinc, copper is widely used as a structural or catalytic cofactor for a diverse range of enzymes in mammals, aiding in cellular respiration, energy metabolism, iron homeostasis, ROS detoxification, and signaling in eukaryotic organisms [24]. Copper has the ability to switch between cuprous ion (Cu^+^) and cupric (Cu^2+^) oxidation states, conferring to cuproenzymes the ability to catalyze redox reactions [25]. With the trivalent form + 3 (Cu^3+^) exists, it is of relatively little biological significance [2]. Copper is a prosthetic group of complex IV (CIV) cytochrome c oxidase, one of the four components of the electron transport chain (ETC) in the process of TCA [25–27]. The ETC is a vital part of the inner mitochondrial membrane (IMM) that generates the proton motive force (PMF), empowering mitochondria to regulate cell metabolism and fate [28]. The proper assembly and function of ETC is dependent on copper. Additionally, copper directly aids in relieving ETC-generated ROS as a cofactor of the copper/zinc superoxide dismutase (SOD1), a protein located in both the cytosol and mitochondrial inner membrane space.

**Fig. 1:**
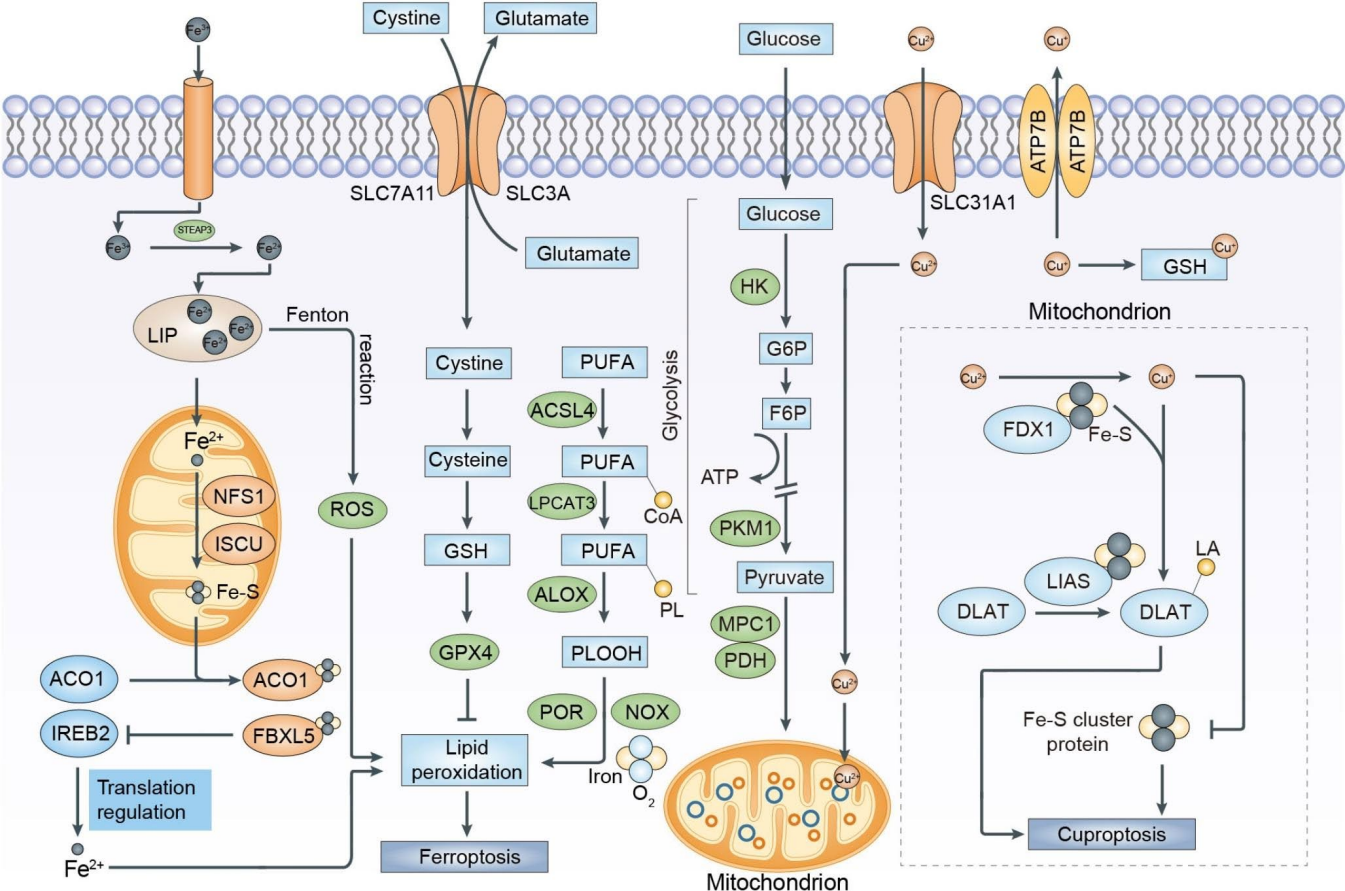
Major Regulatory Pathways in Ferroptosis and Cuproptosis. The regulatory pathways involved in ferroptosis can be roughly divided into three categories. The first one includes the GSH/GPX4 pathway, which encompasses the inhibition of system Xc¯ and glutamine pathway. The second one involves the regulation of iron metabolism, including the modulation of iron-regulatory proteins (IRPs) ACO1 and IREB2 related to ferritin metabolism and the regulatory pathways of FINO2 that impact iron levels. Excessive iron can induce ferroptosis by promoting reactive oxygen species (ROS) production through the Fenton reaction. The third category includes pathways related to lipid metabolism, such as acyl-CoA synthetase long-chain family member 4 (ACSL4), lysophosphatidylcholine acyltransferase 3 (LPCAT3), arachidonate lipoxygenases (ALOXs), etc., which effect lipid regulation and ferroptosis. Excess copper accumulation causes cuproptosis mainly through FDX1-mediated mitochondrial proteotoxic stress. On one hand, FDX1 reduces Cu^2+^ to Cu^+^, facilitating the lipoylation (LA) and aggregation of enzymes, especially dihydrolipoamide S-acetyltransferase (DLAT) involved in the regulation of mitochondrial TCA cycle. On the other hand, FDX1 causes the destabilization of Fe–S cluster proteins. Cu importers (SLC31A1) and exporters (ATP7A/B) regulate cuproptosis sensitivity by affecting intracellular Cu^+^ levels. GSH acts as a thiol-containing copper chelator that can block cuproptosis

Although metal ions are necessary for cellular viability and growth, excessive accumulation of these ions can lead to poisoning and cell death. Various types of cell death can occur in response to different stresses, particularly oxidative stress. RCD involves tightly regulated signaling pathways and specific effector mechanisms, centered around cell death executioner proteins [29]. In contrast, metal-dependent cell death, including ferroptosis and cuproptosis, differs from other forms of RCD. When present in excess, ferrous ions and cuprous ions can be harmful due to their multiple roles in metabolism, resulting in cell damage and cell death. These effects are particularly pronounced in the liver, heart, pancreas, thyroid, and central nervous system. The major regulatory pathways of ferroptosis and cuproptosis are showed in the Fig. 1.

## Ferrous overload plays a key role in ferroptosis induction

### Ferroptosis

Ferroptosis, a form of RCD triggered by iron-dependent lipid peroxidation on cellular membranes, was first identified and named as a distinct phenomenon by Dixon et al. [30] a decade ago. Morphologically and mechanistically, ferroptosis differs from apoptosis, necroptosis, and other types of cell death as outlined in Table 1 [30]. Unlike apoptosis, cells undergoing ferroptosis do not exhibit chromatin condensation and apoptotic body formation, but rather shrunken mitochondrial cristae [31, 32]. The execution of ferroptosis depends on three essential conditions, which are the main prerequisites for driving ferroptosis: (i) synthesis of polyunsaturated fatty acid (PUFA)-containing phospholipids (PLs) (PUFA-PLs) and iron-catalysed peroxidation which is the crux of ferroptosis execution; (ii) regulation of iron metabolism, which initiates the Fenton reaction and acts as an essential cofactor for enzymes that participate in lipid peroxidation; and (iii) modulation of mitochondrial metabolism, which drives ferroptosis through the multidimensional functions of mitochondria in bioenergetics, biosynthesis and ROS generation [15] (Fig. 1). This iron-dependent phospholipid peroxidation is regulated by various cellular metabolic ways, including reductive/oxidative (redox) homeostasis, iron metabolism, mitochondrial activity, metabolism of amino acid and lipid, and glycometabolism [33]. Among these pathways, iron metabolism plays a crucial role in the regulatory network of ferroptosis.

### Iron and its metabolism

Iron is primarily obtained through dietary absorption and recycling in the body [30]. There are two forms of iron that can be absorbed from food: non-heme iron, which mainly exists as insoluble Fe^3+^ and needs to be reduced to Fe^2+^ before absorption and heme iron, which is mostly Fe^2+^ and can be directly absorbed by epithelial cells in the intestinal mucosal [34, 35]. The majority of iron is recycled from phagocytosis of senescent red blood cells, primarily by macrophages in the spleen, bone marrow, and liver [23]. The remaining iron is stored in hepatocytes, contributing to the regulation of systemic iron levels [17]. Iron absorption in duodenal enterocytes is another regulating mechanism for controlling iron levels. The water-soluble Fe^2+^ can be transported to several tissues by binding to transferrin (Tf), after it is transferred from enterocytes into the bloodstream by ferroprotein (FPN) [36]. Transferrin is a plasma protein that binds to iron and delivers it into cells via its receptor 1 (CD71), which is required for iron delivery in most cells and serves as a gatekeeper for regulating iron uptake [37]. Once inside the cell, iron can be utilized for various functions depending on the cellular and systemic conditions. After being released from Tf, iron is transferred to the cytosol by ferrous ion membrane transport protein DMT1 to join the labile iron pool (LIP) [38, 39]. Iron from the LIP can be transferred to mitochondria via DMT1, mitoferrin and siderofexin (SFXN1) [40]. Excess iron from the LIP is stored in ferritin, which can be transferred to and degraded in lysosomes, replenishes the LIP inturn [40]. Hepcidin, a 25 amino acid peptide, is the primary regulatory molecule of systemic iron homeostasis [41]. It binds to ferroportin, triggering the internalization, ubiquitination, and subsequent lysosomal degradation of the iron exporter, thereby reducing available circulating iron [42]. A relatively stable pool of labile iron is normally maintained through the organized regulation of iron absorption, storage, utilization, and export in cells [43]. It is evident that iron overload, which results from imbalanced regulation of iron metabolism, can trigger ferroptosis [15].

### Iron ion overload triggers lipid peroxidation

Although the precise mechanisms by which iron contributes to ferroptosis are not yet fully understood, it is evident that iron overload is a crucial factor in this process. Recent studies have demonstrated that excessive iron ions can induce ferroptosis not only by triggering the Fenton reaction and causing direct peroxidation of PUFA-PLs [44], but also by serving as a necessary cofactor for enzymes involved in lipid peroxidation [45].

The non-enzymatic, iron-dependent Fenton reaction is a crucial step in the process of ferroptosis [13, 46]. Henry John Horstman proposed the reaction in which iron salts react with peroxides to produce hydroxyl radicals (Fe^2+^ + HOOH → Fe^3+^ + OH^–^ + OH•), which now bears his name [47]. This reaction utilizes both iron and oxygen to catalyze a chain reaction, resulting in the propagation of phospholipid peroxidation, which is the hallmark of ferroptosis, leading to the formation of phospholipid hydroperoxides (PLOOHs) [33].

It should be noted that during the initiation phase of this iron-catalyzed chain reaction Fe^2+^ is likely to be more dominant, due to the poor solubility and limited bioavailability of Fe^3+^ in cells [33]. Fe^2+^ acts as a cofactor in the catalytic centers of many important enzymes that are causes for ferroptosis, several of these iron-containing enzymes can promote the lipid peroxidation that drives ferroptosis. For instance, 15-lipoxygenase can complex with phosphatidylethanolamine (PE) binding protein 1 (PEBP1), switching the substrate specificity of the enzyme from free PUFAs to PUFA tails of PLs [48]. Additionally, 12-lipoxygenase is required for p53-dependent ferroptosis [49]. While cytochrome P450 oxidoreductase (POR) and the NADPH oxidases (NOXs) also contribute to ROS production for lipid peroxidation during ferroptosis [50].

FINO2, the fourth class of ferroptosis-inducing compounds, was discovered in 2016 [51]. FINO2 represents an additional means of inducing ferroptosis, as it does not inhibit system Xc¯, deplete glutathione (GSH), directly inhibit glutathione peroxidase 4 (GPX4), or induce GPX4 degradation [15]. One possible mechanism by which FINO2 induces ferroptosis is through the oxidation of Fe^2+^ to Fe^3+^ via Fenton reaction, which generates alkoxyl radicals that initiate lipid peroxidation [15]. Another possible mechanism is that FINO2 binds to and activates lipoxygenases or other iron-dependent enzymes by oxidizing the non-heme iron cofactor; the active form of lipoxygenases is the ferric iron form [52].

Additional mechanisms that regulate cellular iron levels can affect the sensitivity of cells to ferroptosis: exporting iron through ferroprotein or ferritin-containing multivesicular bodies (MVBs) mediated by prominin-2 (prom2), as well as exosomes, can confer resistance to ferroptosis by reducing the intracellular pool of iron and its capacity to promote lipid peroxidation [53]. Ferrous iron is maintained in cells as the labile iron pool, which is bound to low molecular weight compounds, including GSH. The depletion of GSH can not only inactivate GPX4 but also mobilize Fe^2+^ for Fenton chemistry, promoting lipid peroxides propagations and ultimately leading to ferroptosis. In addition, iron storage in ferritin requires the formation of the GSH-iron complex, which is delivered to ferritin via the chaperone poly(rC) binding protein 1 (PCBP1) [54]. To sum up, many genes/proteins are involved in iron metabolic pathways which are involved in the execution of ferroptosis. A more extensive list of the main drugs and compounds implicated in ferroptosis is reported in Table 2.

**Table 2:** Ferroptosis inducers and inhibitors targeting iron homeostasis

Drugs and compounds	Mechanism(s) of action	Effect	References
Erastin	VDACs: Impact on iron metabolism	Facilitate ferroptosis	[31]
	System Xc-: Inhibition of GSH/GPX4 axis		
	ACSL4: Promotion of lipid peroxidation		
RSL-5	VDACs: Impact on iron metabolism		[31]
FINO2	GPX4: Inhibition of GSH/GPX4 axis		[125]
	Ferrous iron: Impact on iron metabolism		
Artemisinin	Lysosomal iron: Increase free iron level		[126]
Withaferin	GPX4: Inhibition of GSH/GPX4 axis		[127]
	HMOX1: Impact on iron metabolism		
Formosanin C	Activates ferritinophagy to increase iron level		[128]
Quercetin	Lysosomal iron: increase free iron level		[129]
Siramesine	Ferroportin, TF: Increased cellular iron		[127]
Lapatinib	Ferroportin, TF: Increased cellular iron		[127]
Deferiprone	Iron: Impact on iron metabolism (chelation)	Suppress ferroptosis	[130]
Deferoxamine	Iron: Impact on iron metabolism (chelation)		[131]
	System Xc-: Impact on GSH/GPX4 axis		
	GPX4: Restoration of GSH/GPX4 axis		
2,2’-pyridine	Iron: Impact on iron metabolism (chelation)		[132]
Ciclopirox	Iron: Impact on iron metabolism (chelation)		[133]

*ACSL4: Acyl-CoA Synthetase Long Chain Family Member 4; GPX4: Glutathione peroxidase 4; GSH: Glutathione; HMOX1: Heme oxygenase 1; TF, transferrin; VDAC: Voltage-dependent anion channel*

## Copper ion overload involved in cuproptosis

### Cuproptosis

Cuproptosis is a distinct form of copper-induced regulated cell death, which was recently reported by Tsvetkov et al. in 2022 [16]. According to their findings, intracellular copper accumulation results in the aggregation of mitochondria lipoylated proteins and destabilization of Fe-S cluster proteins, thereby triggering cuproptosis. This novel mode of Cu-dependent, mitochondrially induced cell death differs from other oxidative stress-induced cell death, such as apoptosis, ferroptosis and necroptosis (Table 1) [16, 55–57]. Unlike these pathways, cuproptosis is triggered by mitochondrial stress, particularly the aggregation of lipoylated mitochondrial enzymes and the loss of Fe-S cluster proteins. Although the detailed mechanism of cuproptosis remains unclear, three critical processes have been identified to play a crucial role in its initiation. These include direct copper binding to lipoylated TCA cycle proteins, lipoylated DLAT oligomerization, and Fe-S cluster proteins destabilization with the ETC complex [55]. Furthermore, seven key genes that promote cuproptosis, with ferrodoxin-1 (FDX1) being the most significant have identified by using genome-wide CRISPSR-Cas 9 loss-of-function screens. FDX1 acts as the primary regulator of copper ionophore-induced cell death, which can reduce Cu^2+^ to its more toxic form Cu^+^. The remaining six genes encode either elements in the lipoic acid pathway (LIPT1, LIAS and DLD) or targets of lipoylation (DLAT, PDHA1 and PDHB).

### Copper and its metabolism

Copper is an essential trace element as a cofactor for a diverse array of enzymes, critical for normal physiological function across various organisms, ranging from bacteria to human cells [11, 25, 58]. Given that copper cannot be synthesized or degraded through metabolic pathways, it must be acquired from external sources [59]. In the body, copper is predominantly stored in the liver, which serves as the main site for copper accumulation and distribution via the bloodstream or biliary excretion. The cellular of copper uptake in mammalian cells is predominantly mediated by CTR1 (SLC31A2), a copper transport protein that exhibits high affinity for the plasma membrane [60]. Although DMT1 (SLC11A2), a divalent metal transporter, has been implicated in copper uptake in certain cultured cell lines, its role in dietary copper absorption is not crucial, as demonstrated by the enterocyte-specific deletion of DMT1, which has no effect on intestinal copper absorption [61]. Intracellular copper is transported to the antioxidant copper chaperone 1 (ATOX1) and CuL (a low molecular weight copper ligand) before being transported into mitochondria, specifically to copper chaperone of cytochrome C oxidase (COX17) and copper chaperone of SOD1 (CCS1) [62]. Despite being an essential cofactor for all organisms, prolonged or chronic exposure to copper can lead to toxicity.

### Copper overload and cuproptosis

Excessive copper ions can participate in the TCA cycle under the influence of FDX1, inducing DLAT aggregation and aberrant mitochondrial protein folding (Fig. 1). Subsequently, the loss of Fe-S protein leads to cell death due to energy metabolism defects [55]. Additionally, copper ion overload can result in redox imbalance, where the antioxidant defense of GSH and SOD is insufficient to counteract the damaging effects of ROS [63].

Cu^2+^ is reduced to Cu^+^ after enters into the mitochondria and then accompanied by the generation of ROS. Unlike ferroptosis, ROS generation is not essential for cuproptosis. Instead, Cu^+^ directly binds to lipoylated proteins and induces the oligomerization of lipoylated DLAT [16]. Tsvetkov et al. [16] discovered that Cu^+^ binding to lipoylated TCA cycle proteins does not simply lead to loss of function but can also result in toxic gain of function. They also found that elesclomol-mediated toxicity in cancer cells was linked to FDX1 levels, elevated mitochondrial respiration rate, and was dependent on Cu availability. Copper overload can also lead to the loss of Fe-S cluster proteins in an FDX1-dependent manner [16]. Fe-S cluster proteins play an important role in life activities, including electron chain transmission, genome stability maintenance, and gene expression regulation [64]. The hydrophilic antioxidant GSH can block the toxicity of Cu^+^ by chelating intracellular Cu, and depletion of glutathione can result in copper-dependent cell death [16].

Excess Cu^+^ can selectively disrupt a set of metabolic enzymes involved in protein lipoylation, which involves attaching the small sulfur-containing metabolite lipoic acid to a substrate protein, enabling catalysis by swinging between different subunits within an enzyme complex, and donating or accepting electrons [56]. Lipoylation is unique to TCA cycle enzymes, occurring in only 4 multimeric metabolic enzymes, all of which are found in the mitochondria, including dihydrolipoamide S-acetyltransferase (DLAT), a subunit of the pyruvate dehydrogenase complex [65]. Copper toxicity can be suppressed by genetically disrupting enzymes required for the synthesis of lipoic acid or of individual lipoylated enzymes themselves [56].

## Metabolism is the core regulator in ferroptosis and cuproptosis

Metabolism pathways play a crucial role in regulating ferroptosis and cuproptosis, despite the distinct mechanisms underlying these two types of metal-dependent cell death. Proteins, lipids, and energy production as well as mitochondrial metabolism, all contribute to the regulation of these processes.

### GSH suppresses ferroptosis and cuproptosis through different ways

Abnormal protein metabolism is crucial in the regulation of ferroptosis and cuproptosis, both of which are influenced by GSH. GSH is a thiol-containing tripeptide synthesized from glycine, glutamate, and cysteine, with cysteine availability being the main limiting factor [66, 67]. GSH exists in two states: reduced (GSH) and oxidized (GSSG) [35]. Reduced GSH is used by GPX4 to reduce reactive PUFA phospholipid hydroperoxides (PUFA-PL-OOH) to non-reactive and non-lethal PUFA phospholipid alcohols (PUFA-PL-OH), which ultimately suppresses ferroptosis. In addition, GSH can block cuproptosis by chelating intracellular Cu [55].

The GSH/GPX4 antioxidation system plays an important role in protecting cells from ferroptosis, which is regulated by the cystine/glutamate transporter (also known as system Xc¯) upstream. The system Xc¯ imports cystine into cells with a 1:1 counter-transport of glutamate. Once in cells, cystine can be oxidized to cysteine, which is used to synthesize GSH in a reaction catalyzed by glutamate–cysteine ligase (GCL) and glutathione synthetase (GSS). GSH, which acts as a reducing cofactor, using by GPX4, can reduce lipid hydroperoxides to lipid alcohols [68]. Depleting GSH can sensitize cells to ferroptosis. For example, the multidrug resistance gene MDR1 drives increased sensitivity to ferroptosis by causing efflux of GSH [69]. The cysteine catabolic enzyme cysteine dioxygenase 1 (CDO1) promotes ferroptosis by depleting cysteine and, in turn, GSH [70]. Synthesizing GSH can contribute to ferroptosis resistance, as shown in a recent report [71]. Glutamate-cysteine ligase, which synthesizes GSH, restrains ferroptosis not only by synthesizing GSH but also by limiting levels of glutamate through its conversion to gama-glutamyl peptides. Thus, the increased levels of glutamate can promote ferroptosis [71]. The role of GSH in suppressing cuproptosis differs from its role in suppressing ferroptosis. GSH chelates intracellular Cu to block cuproptosis. According to the study of Tsvetkov et al. [16], depletion of GSH by buthionine sulfoximine (BSO), a potent inhibitor of the enzyme gamma-glutamyl-cysteine synthetase, increased susceptibility to cuproptosis in A549 lung cancer cells by suppressing lipoylation and promoting DLAT oligomerization [72].

### Destabilization of iron-sulfur cluster proteins promotes cell death

Fe-S clusters are widely distributed and constituted the largest class of metalloproteins across diverse biological systems. These clusters exist in various structures, which the most common being [2Fe-2 S], [3Fe–4 S] or [4Fe–4 S] [73]. Fe-S clusters have a broad range of biological role, including electron transfer within the mitochondrial respiratory chain, the generation of mitochondrial ROS, and DNA metabolism [73–77]. Additionally, Fe-S clusters also play a regulatory role in gene expression in response to oxidative stress, oxygen levels, and iron levels [78–80].

As a critical cofactor in redox maintenance and iron homeostasis, Fe-S clusters and their associated regulatory pathways contribute to ferroptosis evasion in cells by reducing the LIP. The activation of iron-responsive element (IRE)-binding proteins 1 and 2 (IRP1 and IRP2), which are the main regulators of cellular iron pool, occurs when cellular iron levels are low, leading to a rheostat-like iron-starvation response [81]. This response regulates iron uptake, storage, export and usage to increase the available iron pool. IRP1 is modulated by the occupancy of Fe-S clusters and can function as an iron-responsive protein when it loses its Fe-S clusters [82]. Disturbances in Fe-S clusters metabolism are thought to abnormally activate the iron-starvation response via IRP1, overloading cellular iron pools and triggering ferroptosis [46, 83, 84]. Several mitochondrial proteins including cysteine desulfurase (NSF1) [84, 85], iron-sulfur cluster assembly (ISCU) [86], CISD1 [87] and CISD2 [88] inhibit ferroptosis by increasing the biosynthesis of Fe-S. In contrast, frataxin, another protein involved in Fe-S clusters assembly, is upregulated in different cancer types and promotes cancer cell resistance to ferroptosis [89].

Excess copper ions inhibited the synthesis of Fe-S clusters under the regulation of FDX1, leading to a reduction in Fe-S cluster proteins [16]. Knockdown of FDX1 causes the loss of protein-lipid acylation, decreased mitochondrial respiration, accumulation of pyruvate and 𝛼-ketoglutarate, and loss of iron-sulfur cluster proteins [16]. However, the relationship between the reduction of Fe-S cluster proteins and cuproptosis remains to be explored. Additionally, protein aggregates could trigger cell death by disrupting the function of mitochondrial enzymes involved in the synthesis of iron-sulfur clusters, which are essential cofactors for other enzymes [64].

### Lipid peroxidation, a hallmark of ferroptosis

Lipid peroxidation is a key feature of ferroptosis and affects PUFAs. Initially, it was believed that free PUFAs were the driving force behind ferroptosis. However, several studies have shown that free fatty acids are not the direct drivers of ferroptosis. Instead they must be esterified into membrane phospholipids and oxidized to transmit the ferroptosis signal. Specifically, PE containing arachidonic acid (AA) or its derivative adrenaline has been identified as the key phospholipid that induces ferroptosis. Acyl-coenzyme A (Acyl-CoA) synthetase long-chain family member 4 (ACSL4) and lysophosphatidylcholine acyltransferase 3 (LPCAT3) are involved in the biosynthesis and remodeling of PE, activating PUFAs and influencing the transmembrane characteristics of PUFAs [90, 91]. Therefore, reducing the expression of ACSL4 and LPCAT3 can decrease the accumulation of lipid peroxide substrates in cells, leading to the inhibition of ferroptosis. Finally, PUFA-PE can undergo further oxidative reactions under the catalysis of lipoxygenases (ALOXs) and ultimately induce ferroptosis [45].

### Glucose metabolism governs cell death through the energy regulation

The role of glucose metabolism in regulating ferroptosis and cuproptosis has not been extensively studied, unlike lipid and amino metabolism. Iron is essential in glucose metabolism, and both iron deficiency and overload can affect glucose metabolism. Iron overload is associated with decreased insulin sensitivity and promoted insulin resistance due to reduced glucose uptake, likely caused by increased ROS production and impaired autophagy [92].

Glucose is the primary fuel of the mitochondrial TCA cycle, and it has been shown to regulate ferroptosis [33]. Studies by Lee et al. [93] and Li et al. [94] have suggested that glucose starvation may suppresses ferroptosis due to the activation of AMP-activated kinase (AMPK) signaling, rather than modulation of TCA and mitochondrial respiration. AMPK activation leads to impaired biosynthesis of PUFAs, which are essential for ferroptosis, resulting in a protective effect against cell death [33, 45, 95]. Glucose starvation, however, can also increase ROS production, implying that it promotes ferroptosis [96]. Although the regulatory role of glucose metabolism in cuproptosis is not clear, cells that rely on mitochondrial respiration are more sensitive to copper ionophores, suggesting that cuproptosis is regulated by mitochondria respiration rather than glycolysis [16]. Further studies are needed to explore the details of how aerobic OXPHOS of glucose regulates cuproptosis.

## Pathological contexts of kidney Diseases related to ferroptosis and cuproptosis

Metal ions are micronutrients that play critical roles in cellular functions and metabolic processes in the human body. However, excessive accumulation of metal ions can result in the generation of ROS, leading to cell death, tissue damage, and the development of various kidney diseases, including acute kidney injury, fibrosis, renal cell cancers and ischemia reperfusion injury of kidney transplantation.

### Acute kidney injury

Acute kidney injury (AKI) is a common and serious clinical renal syndrome, which is caused by a variety of pathogenic factors and is an urgent disease to be solved. Recently, evidence has showed that ferroptosis plays an important role in AKI.

Studies have found many mechanisms by which iron death affects acute renal injury. ACSL4 is a promotor for ferroptosis and is negatively regulated by HIF-1a [97] but activated by HMGB1 [98]. When it comes to the ischemia reperfusion injury, HIF-1α downregulated and HMGB1 released from epithelial cells which leads to higher ACSL4 expression. Besides, ACSL4 is correlated with the infiltration of macrophages and that neutrophils are recruited by ferroptotic-cell induced macrophages. Lack of FSP1 or targeted manipulate the center of the selenoprotein glutathione peroxidase 4 (GPX4cys/-) makes it sensitive for tubular to ferroptosis and causes a special tubular necrosis [99]. Tripartite motif containing 21 (TRIM21) highly expresses in kidney with ischemia reperfusion injury (IRI) and affects ferroptosis by inducing ubiquitination degradation of GPX4 [100]. Polymyxin B (PMB) is widely used in multi-resistant gram-negative infections while its side-effects involve acute kidney injury. Recent study identified that PMB causes AKI by promoting ferroptosis with RNA-sequencing and further explored the mechanism that PMB decreases the expression of SLC7A11 and increase TFR1 through the activation of P53 [101]. On the other hand, some targets are capable of reversing ferroptosis. Activated Farnesoid X receptor (FXR) prevents against AKI by modulating transcription of ferroptosis-associated genes, like AIMD2, GGT6 and GSTA4 [102]. BNIP3 and PINK1-PINK2 related-mitophagy protect kidney from cisplatin-induced acute injury through ROS/HO1/GPX4 pathway [103].

In view of the prominent role of iron death in AKI, more and more studies have begun to try and develop drugs to inhibit ferroptosis to alleviate AKI. Quercetin decreases malondialdehyde and ROS and increases the level of GSH which turn to the blockage of morphologic changes of ferroptotic cells [104]. Vitamin K1 has been identified as an exciting inhibitor for ferroptosis and is prepared for AKI treatment [105]. Irisin alleviates AKI through activation of SIRT1/NRF2 pathway [106]. Leonurine protects kidney from cisplatin-induced injury by activating Nrf2/NRF2 pathway [107]. Paeoniflorin (PF) is a traditional Chinese medicine that protects kidney from AKI. Ma et al. identified in IRI model that PF prevents kidney from ferroptosis-induced injury by upregulating SLC7A11 [108].

### Kidney fibrosis

Ferroptosis promotes interstitial fibrosis and inflammation in chronic kidney diseases or UUO or I/R mouse models. Ferroptosis inhibition ameliorated kidney fibrosis by reducing MCP-1 excretion and chemotaxis of macrophages [109]. Combination of Melatonin and Zileuton synergistically protect kidney from fibrosis in UUO model by upregulating AKT/mTOR/NRF2 signaling pathway which inhibits ferroptosis [110]. Formononetin inhibits Smad3/ATF3/SLC7A11 pathway, increases the expression of SLC7A11 and GPX4 and promotes Nrf2 nuclear accumulation, resulting in the amelioration of kidney fibrosis and ferroptosis [111].

### IRI after kidney transplantation

IRI largely affect function recovery after kidney transplantation and studies have found that ferroptosis leads to delayed recovery of renal allograft. It is found that miR-20a-5p could reduce the kidney loss and structural damage by inhibiting ACSL4-dependent ferroptosis [112]. Tang et al. screened ferroptosis-related hub genes (IL-6, ATF3 and JUN) of IRI post-renal transplantation. They could be diagnostic biomarkers and target for preventing dysfunction of transplanted kidney [113].

### Renal cancers

In recent years, it has been found that ferroptosis and other ion death are inhibited in the occurrence and progression of renal tumors. Exploring the mechanism of tumor inhibiting cell ion death and reactivating it may be a promising therapy for tumor treatment.

Clear cell renal cell carcinoma (ccRCC) is a main subtype among renal cancers. Protein disulfide isomerase A4 (PDIA4) confers resistance to ferroptosis via induction of ATF4/SLC7A11 in renal cell carcinoma. And Salinomycin exert anti-tumor ability by inhibiting PDIA4 [114]. The unique metabolic state of ccRCC is related to ferroptosis sensitivity and GPX4 plays a vital role in this progress [115]. Kruppel like factor 2 (KLF2) correlates to the expression of GPX4 in ccRCC and overexpressed KLF2 inhibits tumor growth and invasion by regulating ferroptosis [116]. Acyl-CoA synthetase long-chain family member 3 (ACSL3) regulates the accumulation of lipid droplets in ccRCC and is essential for tumor growth. In addition, ACSL3 also modulates ferroptosis sensitivity in a manner dependent on the composition of exogenous fatty acids. Both functions of ACSL3 could be exploited for ccRCC therapy [117]. AIM2 promotes progression and sunitinib resistance through FOXO3a-ACSL4 axis-regulated ferroptosis [118]. The Hippo Pathway Effector TAZ and ISCA2 are also contribute to ferroptosis in ccRCC [119]. What’s more, Lai et al. developed a predictive model with 8 ferroptosis-related LncRNA predicting prognosis of ccRCC [120]. Combined use of fatty acid amide hydrolase (FAAH) inhibitor URB597 and ferroptosis inducer (1 S,3R)-RLS3 shows strong synergetic inhibition of RCC through induction of G1 cell cycle arrest and promotion of ROS. The dual targets therapy modulates RCC sensitivity to ferroptosis [121].

FDX1, a key factor of cuprotosis, is found to de downregulated in ccRCC which shows correlation between cuproptosis and tumor mechanically [122]. Gang Luo et al. identified 6 cuproptosis-related ferroptosis genes (TRIB3, SLC2A3, PML, CD44, CDKN2A and MIOX) that correlate with worse survival [123]. Besides, they created a prognostic model incorporating these signatures and predict survival.

Renal medullary carcinoma (RMC) is a lethal malignant tumor derived from kidney medulla. It is characterized by the lack of SMARCB1 expression, which is a key subunit of the SWItch/Sucrose Non-Fermentable (SWI/SNF) chromatin remodeling complex. However, the origin of RMC still remains unclear. Recently, Bujamin H Vokshi et al. defined that the loss of transcription factors TFCP2L1, HOXB9 and MITF and the switch on of MYC and NFE2L2-related oncogenic and ferroptosis resistance programs involved in the convert from thick ascending limb (TAL) to RMC cells. And the transcription switch is reversed by SMARCB1 and leads to ferroptotic cell death. Ferroptosis resistance correlates TAL cells survival with high extracellular medullar iron concentrations with character of sickle cell, which possibly is an explanation for the reason why RMC is the only epithelial cell-derived SMARCB1-lost tumor [124].

## Perspective and conclusion

To fully understand ferroptosis and cuproptosis, it is important to consider the following key points.

First, these are distinct forms of metal-dependent cell death that differ from other regulated programmed cell death. Ferroptosis is characterized by unique morphological and biochemical features, such as mitochondrial shrinkage and the accumulation of reactive oxygen species (ROS). In contrast, the exact changes in morphological and biochemical features of cuproptosis are still unclear.

Second, the accumulation of metal ions is not synonymous with ferroptosis or cuproptosis, as both iron and copper can have numerous effects beyond including cell death. The oxidation state of metal is also important, with Fe^2+^ promoting ferroptosis, whereas Fe^3+^ is generally inert and stored in ferritin. Similarly, Cu^+^ is a more toxic form than Cu^2+^ in promoting cuproptosis. Therefore, it is important to determine the redox state of these two metals and whether they contribute specifically to ferroptosis and cuproptosis in a given context.

Third, the production of ROS through iron-dependent Fenton reaction is a key mechanism that propagates lipid peroxidation, driving ferroptosis. ROS can also be generated from copper-based Fenton reaction, but it is unclear whether this is involved in ferroptosis.

**Fig. 2:**
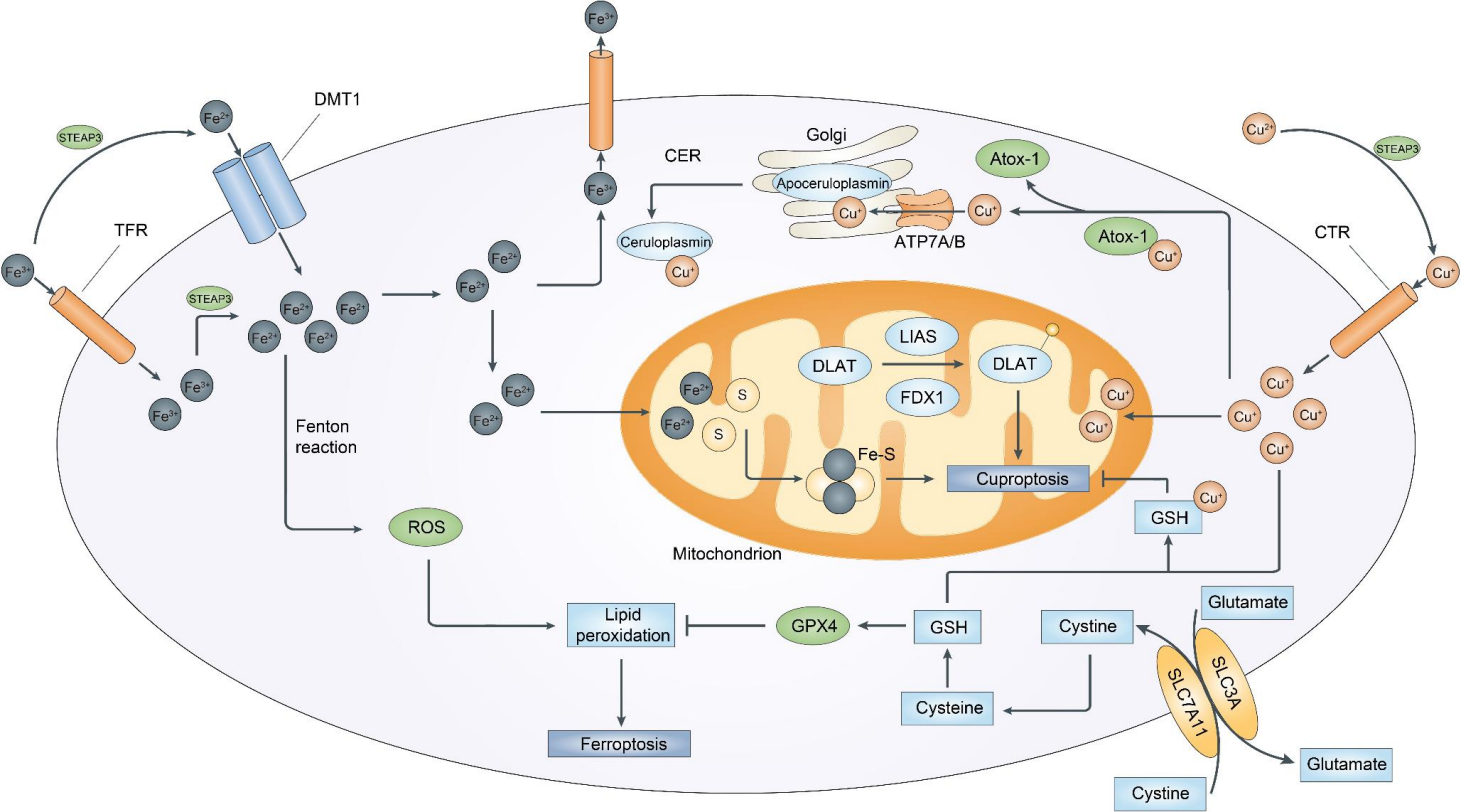
Crosstalk Between Ferroptosis and Cuproptosis in the Context of Ion metalbolism. The interactions between cuproptosis and ferroptosis can be categorized into three main groups, considering the close relationship between copper and iron metabolism. First, copper is an essential cofactor for several enzymes and proteins, such as ceruloplasmin, which is responsible for iron export. It can oxidize Fe^2+^ to Fe^3+^, thus playing a role in iron regulation. Second, under conditions of copper overload, ROS production from copper-base Fenton reaction can increase. Third, GSH is the principal substrate for GPX4, and the GSH synthase inhibitor has similar cytotoxicity in cells supplemented with either copper or iron. Additionally, destabilization of Fe–S cluster proteins can also contribute to cuproptosis

Finally, both ferroptosis and cuproptosis affect mitochondria but have some difference. Mitochondria can act as initiators and amplifiers of ferroptosis, while copper-dependent cell death is dependent on mitochondrial respiration. Nonetheless, our understanding of these forms of cell death remains incomplete, and the interactions between cuproptosis and ferroptosis need to be explored, given the close relationship between copper and iron metabolism (Fig. 2). (1) Copper is an essential cofactor for several enzymes and proteins, such as ceruloplasmin, which is responsible for iron export. It can oxidize Fe^2+^ to Fe^3+^, and is involved in the regulation of iron. When there is an excess of copper, ferroptosis may be inhibited. (2) Conversely, under conditions of copper overload, the ROS from copper-base Fenton reaction can increase, which may sensitize cell to ferroptosis. (3) Copper deficiency can inhibit iron absorption, transport and hemoglobin synthesis. (4) in cells of low Fe levels, the labile Cu^+^ content increases significantly. Even if iron intake is normal, a copper deficiency in the diet can cause anemia. (5) GSH is the principal substrate for GPX4, and the GSH synthase inhibitor BSO has similar cytotoxicity in cells supplemented with either copper or iron. These observations suggest a possibility of crosstalk between cuproptosis and ferroptosis in downstream events. Furthermore, it is still unclear whether other forms of cell death are involved in ferroptosis and cuproptosis. Future studies should aim to identify the details of metal-induced cell death and investigate whether other trace metal ion-induced forms of cell death exist.

In summary, there are many opportunities to elucidate the mechanisms of metal ions overload in both ferroptosis and cuproptosis. Such studies will illuminate the breadth of physiological and pathological roles related to ion overload of cell death. Moreover, targeting only one form of cell death, either ferroptosis or cuproptosis, may cause an imbalance in another metal ion. Therefore, it is important to consider the interactions between iron and copper overload when developing novel ferroptosis-or cuproptosis-based therapies for clinical use in the future.

## Data Availability

Not applicable.
